# Xrn1/Pacman affects apoptosis and regulates expression of *hid* and *reaper*

**DOI:** 10.1242/bio.201410199

**Published:** 2015-04-02

**Authors:** Joseph A. Waldron, Christopher I. Jones, Benjamin P. Towler, Amy L. Pashler, Dominic P. Grima, Stephen Hebbes, Samuel H. Crossman, Maria V. Zabolotskaya, Sarah F. Newbury

**Affiliations:** Brighton and Sussex Medical School, University of Sussex, Brighton BN1 9PS, UK

**Keywords:** Apoptosis, Compensatory proliferation, RNA stability, Wing imaginal discs, XRN1

## Abstract

Programmed cell death, or apoptosis, is a highly conserved cellular process that is crucial for tissue homeostasis under normal development as well as environmental stress. Misregulation of apoptosis is linked to many developmental defects and diseases such as tumour formation, autoimmune diseases and neurological disorders. In this paper, we show a novel role for the exoribonuclease Pacman/Xrn1 in regulating apoptosis. Using *Drosophila* wing imaginal discs as a model system, we demonstrate that a null mutation in *pacman* results in small imaginal discs as well as lethality during pupation. Mutant wing discs show an increase in the number of cells undergoing apoptosis, especially in the wing pouch area. Compensatory proliferation also occurs in these mutant discs, but this is insufficient to compensate for the concurrent increase in apoptosis. The phenotypic effects of the *pacman* null mutation are rescued by a deletion that removes one copy of each of the pro-apoptotic genes *reaper*, *hid* and *grim*, demonstrating that *pacman* acts through this pathway. The null *pacman* mutation also results in a significant increase in the expression of the pro-apoptotic mRNAs, *hid* and *reaper*, with this increase mostly occurring at the post-transcriptional level, suggesting that Pacman normally targets these mRNAs for degradation. Our results uncover a novel function for the conserved exoribonuclease Pacman and suggest that this exoribonuclease is important in the regulation of apoptosis in other organisms.

## INTRODUCTION

Apoptosis, or programmed cell death, is crucial to normal embryonic development and metamorphosis of multicellular organisms, as well as being important in disease. Control of apoptosis is coordinated with that of proliferation, with many signalling pathways implicated in the normal control of tissue growth involved in both processes (Danial and Korsmeyer, 2004). The key components of apoptosis pathways are well known and highly conserved, and many of the signalling pathways that regulate apoptosis have been elucidated (Domingos and Steller, 2007; Fuchs and Steller, 2011; Hay and Guo, 2006; Salvesen and Abrams, 2004; Steller, 2008; Xu et al., 2009). Although post-transcriptional processes that work at the level of RNA stability are known to be important in a number of cellular processes [e.g. inflammation (Sanduja et al., 2011)] their contribution in the control of apoptosis are not well understood.

*Drosophila* provides an excellent model system for the study of apoptosis because of its genetic tractability and the similarities of its apoptosis pathways to that of other organisms. In *Drosophila*, the regulation of caspase activation is a major strategy by which apoptosis is regulated. Factors inducing apoptosis, such as developmental signals or irradiation, result in activation of pro-apoptotic proteins such as Reaper, Hid and Grim. These proteins then trigger ubiquitin-mediated degradation of DIAP1 (XIAP), releasing Dronc (Caspase-9-like) from DIAP1 inhibition. Together with the scaffolding protein Ark (Apaf-1), free Dronc proteolytically cleaves and activates the effector Caspase-3-like caspases DrICE (Drosophila interleukin-1-converting-enzyme) and Dcp-1 (death caspase-1). These caspases then trigger downstream events such as DNA fragmentation, inhibition of translation initiation and formation of apoptotic bodies (Domingos and Steller, 2007; Thomas and Lieberman, 2013).

This paper describes the discovery of a new player in the control of apoptosis, namely the Pacman/Xrn1 exoribonuclease. Pacman (Pcm) is a highly conserved processive 5′-3′ exoribonuclease that degrades mRNAs after they have been decapped (Garneau et al., 2007; Jones et al., 2012; Nagarajan et al., 2013). As well as being a key enzyme in RNA turnover, XRN1 homologues are also involved in nonsense-mediated decay (NMD) and degradation of mRNAs after they have been targeted by small interfering RNAs or miRNAs (Orban and Izaurralde, 2005). In *Drosophila*, mutations in *pacman* result in specific phenotypes such as defects in wound healing, epithelial closure and male fertility (Grima et al., 2008; Jones et al., 2013; Till et al., 1998; Zabolotskaya et al., 2008). The Pacman protein is maternally contributed and is differentially expressed during development (Grima et al., 2008).

In this study, we show that the 5′-3′ exoribonuclease Pacman/Xrn1 regulates apoptosis in *Drosophila* wing imaginal discs. We generated a null mutation in *pacman* (*pcm^14^*) and confirmed, using a novel assay, that it has no detectable 5′-3′ exoribonuclease activity *in vivo*. We found that the *pcm^14^* mutation results in small imaginal discs and lethality at the pupation stage. Using mosaic analysis and immunocytochemistry, we show that the small wing imaginal discs result from increased apoptosis, even though compensatory proliferation is also occurring. Finally, we also demonstrate that Pacman acts through the caspase pathway and that the *pcm^14^* mutation causes post-transcriptional upregulation of the pro-apoptotic genes *hid* and *reaper*. This is the first time that a 5′-3′ exoribonuclease has been found to specifically regulate apoptosis. The conservation of Pacman/Xrn1 throughout eukaryotes suggests that it may also regulate apoptosis in other organisms.

## MATERIALS AND METHODS

### Fly stocks and crosses

Fly stocks were cultivated on standard media at 25°C in uncrowded conditions. All the stocks used were from the Bloomington Stock Center unless otherwise stated.

Creation of the *pcm^5^* allele was previously described (Grima et al., 2008); the same methodology was used to create the *pcm^14^* mutant and its corresponding wild-type control (*pcm^WT^*). The *pcm^14^* allele is a 3,501 bp deletion extending from the P-element insertion site towards *pcm*, deleting 3,068 bp into the 3′ of the gene, completely removing exons 7–11 and part of exon 6. The 5′ of the neighbouring non-coding RNA *CR43260* is also deleted. Additionally, a new hypomorphic allele, *pcm^13^*, was created which is a 2,222 bp deletion extending in both directions from the P-element insertion site, deleting 590 bp from the 3′ of *pacman* (including exons 10 and 11), 529 bp from the 3′ of *Nat1* and entirely deleting *CR43260*. Despite the additional deletions of *CR43260* and the 3′ of *Nat1*, the phenotypes observed for *pcm^13^* were weaker than those seen for the *pcm^5^* allele, showing that deletion of *CR43260* does not contribute to the *pacman* mutant phenotypes. For use as a wild-type control for *pcm^13^* and *pcm^14^*, a line from which the P-element was excised without causing a deletion was selected (referred to as *pcm^WT^*). To ensure that the lethality of the *pcm^14^* chromosome was due solely to the deletion at the *pacman* locus, chromosomal crossover was allowed to occur between the *pcm^14^* chromosome (*w^1118^ pcm^14^*) and a chromosome containing multiple recessive markers (*y^1^ cv^1^ v^1^ f^1^ car^1^*, stock 1515). Phenotypes of males carrying recombinant chromosomes were examined and none were found without the *f^1^* and *car^1^* markers, indicating the lethality of the chromosome stemmed from this region, which contains the *pacman* locus. Additionally, a translocation from *X* to *Y*, *T(1;Y)B92* [stock 101110 (*In(1)FM7/T(1;Y)B92, y^1^ y+ B^S^*) from the Kyoto Drosophila Genetics Research Center], which includes the *pacman* locus was able to rescue the lethality of the *pcm^14^* chromosome (*w^1118^ pcm^14^/T(1;Y)B92, y^1^ y+ B^S^* males survived to adulthood).

GAL4 drivers used were *nub-GAL4* (stock 25754; *P{UAS-Dcr-2.D}1, w^1118^; P{GawB}nub-AC-62*), *69B-GAL4* (stock 1774; w*;; P{GawB}69B) and *en-GAL4 UAS-GFP-actin/CyO* (kindly donated by Paul Martin, University of Bristol). w*; *P{tubP-GAL80ts}20*; *TM2*/*TM6B, Tb^1^* was used to inhibit GAL4 activity. To prevent ectopic *DIAP1* expression during embryogenesis the *GAL80^ts^* system was used to inhibit GAL4 activity until the larval stages of development. This was achieved by moving the larvae from 19°C to 29°C 48 hours AEL.

The *UAS-pcm^RNAi^* stock used was stock number 21677; *w^1118^; P{GD10926}v21677* (Vienna *Drosophila* RNAi Centre). Construction of the *UAS-pcm^WT^* construct has been described previously (Grima et al., 2008). *UAS-pcm^ND^* was created using the Stratagene QuikChange mutagenesis kit (cat. no. 200521) to mutate an A to a G residue (GAG→GGA) at the conserved position E177G. Alteration of the homologous residue (E178G) in yeast abolishes 99.9% of exonuclease activity (Page et al., 1998). The entire *pacman* construct was checked by sequencing. Germline transformation of *w^1118^* flies was then carried out using standard methods (Grima et al., 2008).

The *Df(1)ED7452* stock (full genotype *Df(1)ED7452, w^1118^ P{3′.RS5+3.3′}ED7452/FM7i, P{ActGFP}JMR3*) was created by the DrosDel method (Ryder et al., 2004) and was submitted to the Bloomington Stock Center (stock 38466). The source of the *Adh^fn6^* allele was *Adh^fn6^ cn^1^; ry^506^* (stock 1983).

For the mosaic analysis experiment the *w^1118^ pcm^14^* chromosome was recombined with *y^1^ w^1118^ P{neoFRT}19A* (stock 1744) (as in Xu and Rubin, 1993) to give genotype *w^1118^ pcm^14^ P{neoFRT}19A/FM7i, P{ActGFP}JMR3* which was then crossed to P{Ubi-mRFP.nls}1, w*, P{hsFLP}122 P{neoFRT}19A (stock 31418). By selecting against GFP and for RFP, the offspring from this cross used in the experiment were *w^1118^ pcm^14^, P{neoFRT}19A*/P{Ubi-mRFP.nls}1, w*, P{hsFLP}122 P{neoFRT}19A.

In order to inhibit apoptosis the following stocks were used; w^*^; *P{lacW}Ark^k11502^ Ark^82^*/*CyO y^+^*, *Df*(*3L*)*H99 kni^ri-1^ p^p^*/*TM3 Sb^1^* and w^*^;; *P{UAS-DIAP1.H}3*.

### Measurement of wing and wing imaginal disc sizes

Wing imaginal discs were dissected from L3 larvae in Ringer's solution and photographed using a Nikon Digital Sight DS-Fi1 camera mounted on a Nikon SMZ800 dissecting microscope at a constant magnification. The area of each disc was then measured in arbitrary units using ImageJ (http://rsbweb.nih.gov/ij/) and normalised to wild type as a percentage. For images, L3 wing imaginal discs were dissected and mounted in Aqua-Poly/Mount (Polysciences, cat. no. 18606-20). Wings were mounted in DPX medium (Fisher Scientific, cat. no. 10050080) (weighted down overnight). Images were produced using Axiovision 4.7 on an Axioplan microscope (Carl Zeiss).

### Measurement of larval size, development time and survival

Larval surface area was measured essentially as described in (Hou et al., 2012) using a Nikon Digital Sight DS-Fi1 camera mounted on a Nikon SMZ800 dissecting microscope. For survival experiments, larvae of the desired genotype were placed in fresh vials and the number of eclosing adults was counted. Larval development time was measured by placing L1 larvae into food vials and counting pupae as they pupated. For the larval weight experiment, larvae were staged by the addition of bromophenol blue to the food (0.05%) and selecting only larvae that had cleared the dyed food from their gut for weighing.

### Immunocytochemistry

Immunocytochemistry was performed essentially as described in (Sullivan et al., 2000). Images were taken with a Zeiss Axiovert confocal microscope equipped with a LSM520 Meta. Primary antibodies used were anti-Pacman (Grima et al., 2008) (1:500), anti-Cleaved Caspase-3 (Asp175) (Cell Signaling, cat no. 9661;1:400), anti-phosphohistone H3 (Ser10) (Cell Signaling, cat. no. 9701; 1:400) and anti-Wingless (4D4) (Developmental Studies Hybridoma Bank; 1:400). Secondary antibodies used were Cy3-conjugated monoclonal goat anti-rabbit IgG (Jackson ImmunoResearch, cat. no. 711-165-152; 1:400), Cy3-conjugated monoclonal Donkey anti-mouse IgG (Jackson ImmunoResearch, cat. no.715-165-151; 1:400) and FITC-conjugated polyclonal goat anti-rabbit (Sigma, cat. no. F9887; 1:200).

### BrdU incorporation

BrdU incorporation and labelling was performed essentially as previously described (http://theduroniolab.web.unc.edu/files/2013/10/Eye-disc-BrdU.pdf). 5′ bromodeoxyuridine was fed to 120-hour-old larvae at a concentration of 0.1 mg/ml. Wing discs were dissected, fixed in 4% formaldehyde and permeabilised for 45 min in PBS + 0.6% Triton X-100. Discs were incubated in 2N HCl for 30 min at room temperature and then neutralised in sodium borate. Discs were washed 3 times in PBS + 0.3% Triton X-100 before incubation in anti-BrdU overnight at 4°C (Developmental hybridoma bank (G3G4), 1:20). The secondary antibody was Cy3-conjugated monoclonal Donkey anti-mouse IgG (Jackson ImmunoResearch, cat. no.715-165-151; 1:350).

### RNA extraction and qRT-PCR

RNA extractions were performed using a *mir*Vana miRNA isolation kit (Life Technologies, cat. no. AM1560). Samples were treated with a DNA-free kit (Life Technologies, cat. no. AM1906) and their concentrations measured on a NanoDrop 1000 spectrophotometer (Thermo Scientific). For qRT-PCR, cDNA was prepared in duplicate from the RNA samples using a High Capacity cDNA Reverse Transcription Kit (Life Technologies, cat. no. 4368814) with random primers or oligo dT primers as appropriate. A “no RT” reaction was performed in parallel as a control to confirm that all genomic DNA had been degraded. qRT-PCR was performed on each cDNA replicate in duplicate (i.e. 4 technical replicates in all), using TaqMan Universal PCR Master Mix, No AmpErase UNG (Life Technologies, cat. no. 4324018) and an appropriate TaqMan mRNA/pre-mRNA assay (Life Technologies). All mRNA TaqMan assays used were pre-designed. For custom pre-mRNA assays, 100 nt of sequence of the desired target area was submitted to Life Technologies' web-based Custom TaqMan Assay Design Tool as in (Jones et al., 2013) (supplementary material Fig. S3). *RpL32* (*Rp49*) was used for normalisation.

### Western blotting

Western blotting was performed on samples containing 60 wing imaginal discs. Tubulin was used as an internal control. Mouse anti-Tubulin primary antibody (Sigma, cat. no. T9026) was used at a 1:2000 dilution with an anti-mouse-HRP conjugated secondary antibody (Sigma, cat. no. A2304) at 1:80,000. Rabbit anti-Pacman was used at 1:2,000 with an anti-rabbit-HRP conjugated secondary antibody (Sigma, cat. no. 1949) at 1:80,000. Antibody binding was detected using Amersham ECL detection reagents (GE Healthcare, cat. no. RPN2209). Relative quantification of bands was performed in ImageJ.

### Mosaic analysis

48±4 hours old larvae of the desired genotype (see Fly stocks and crosses above) were subjected to heat shock at 37°C for 1 hour. The larvae were then placed at 25°C until L3 larvae had developed. The wing imaginal discs were then dissected in Ringer's solution and mounted on poly-l-lysine treated slides in Aqua-Poly/Mount. Images were taken with a Zeiss Axiovert confocal microscope equipped with a LSM520 Meta.

### Calculating the mitotic/S phase index

To count the number of cells in M phase from the phosphohistone H3 staining, or S phase from the BrdU incorporation, the ImageJ plugin, DeadEasy MitoGlia was used (Forero et al., 2010). All discs were stained and photographed under the same conditions using the standard immunocytochemistry protocol above. All settings were kept as default except the minimum threshold was set to 60. The mitotic/S phase index was then calculated for each disc using the following calculation:
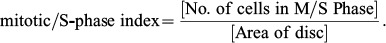


### Statistical analyses

All statistical analyses were performed in GraphPad Prism 6. All data analysed were compatible with parametric tests. Two-sided two-sample *t*-tests were used to compare the means of single test groups to single control groups. If multiple comparisons were required, a one-way ANOVA was performed with a post-test to compare the means of each possible pair of samples.

## RESULTS

### A null mutation in *pacman* results in lethality

**Fig. 1.**
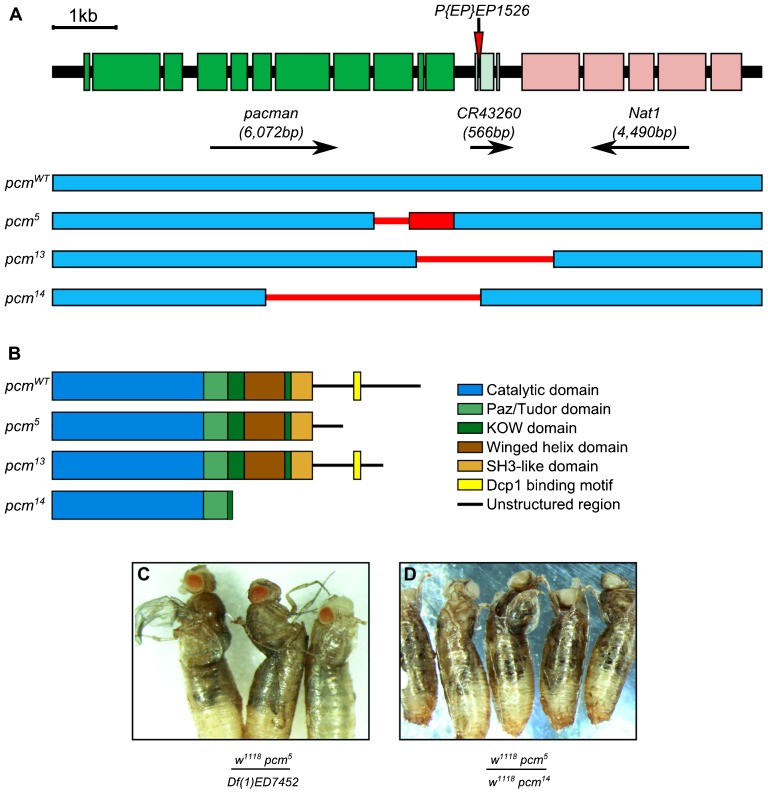
*pcm^14^* is a null allele of *pcm*. (A) The genomic region of the *pacman* gene and the alleles *pcm^5^*, *pcm^13^* and *pcm^14^*, created by imprecise excision of *P{EP}EP1526*. Green boxes represent exons of *pacman*, blue boxes represent wild-type genomic DNA sequences unaffected by the imprecise excisions and thin red lines indicate regions of the genomic DNA that are deleted in each allele. *pcm^5^* is a hypomorphic allele (Grima et al., 2008; Jones et al., 2013) that consists of a 516 bp deletion causing the remainder of the coding region to be out of frame (red box). *pcm^13^* is also a hypomorphic allele consisting of a 2,222 bp deletion extending in both directions from the P-element insertion site. *pcm^14^* is a 3,501 bp deletion extending 3,068 bp into the 3′ of *pacman*, removing exons 7–11 and part of exon 6, as well as the 5′ region of *CR43260*. (B) The domains of Pacman proteins encoded by each allele. (C,D) When cultured at 19°C, *pcm^5^/Df(1)ED7452* flies become stuck when eclosing from their pupal cases. *Df(1)ED7452* is a 17,963 bp deficiency that removes four genes including *pacman*. Similar results are obtained for *pcm^5^/Df(1)JA27* which removes at least 69 genes including *pacman*. The same phenotype is produced when *pcm^5^/pcm^14^* flies are cultured at 19°C, showing that *pcm^14^* is a null allele.

In our previous work, we analysed the phenotypic consequences of a hypomorphic allele of *pcm*, *pcm^5^*. This was found to result in a number of phenotypes including smaller wing imaginal discs and wings (Jones et al., 2013). Since this allele leaves intact a large portion of the Pacman protein (amino acids 1–1264 out of 1612) including the catalytic domain (amino acids 1–674), it was likely that there was still some exoribonuclease activity within the cells of the mutant flies. To fully understand the function of Pacman in development, it was necessary to generate a null allele of *pcm*, to eliminate all the activity of the Pacman enzyme. This was achieved using imprecise excision of the P-element *P{EP}EP1526* to generate a new lethal allele (*pcm^14^*) (Fig. 1A). As expected, the *pcm^14^* allele results in a stronger phenotype than the hypomorphic *pcm^5^* allele in that hemizygous males and homozygous females die during pupation before any adult structures are formed (100% penetrance). To confirm that the lethality was directly caused by a lack of *pacman* expression we made use of the GAL4-UAS system to express wild-type *pacman* cDNA (*UAS-pcm^WT^*) in particular larval tissues, in an attempt to rescue the lethality. Using the *69B-GAL4* driver (which drives expression throughout the wing, eye, haltere and leg imaginal discs and in ectodermal tissue during stages 9–17 of embryogenesis) (Brand and Perrimon, 1993; Flybase Consortium, 1996) the lethality is completely rescued (supplementary material Fig. S1), showing that the lethality of *pcm^14^* stems from the *pacman* locus. We also demonstrated that the *pcm^14^* allele is genetically a null allele as it acts as a deficiency in combination with the *pcm^5^* allele (Fig. 1C,D).

The Pacman protein comprises a N-terminal catalytic domain followed by PAZ/TUDOR, KOW, Winged helix and SH3 domains (Jinek et al., 2011) (Fig. 1B). The C-terminal domain is relatively unstructured and includes short sections of conserved amino acids which bind cofactors such as the decapping factor Dcp1(Braun et al., 2012). The lethal phenotype of Pacman could result from a lack of exoribonuclease activity or a lack of binding to decapping or other factors. To test this we used a nuclease-dead version of Pacman, where the critical glutamate in the conserved magnesium binding site has been mutated to a glycine (E177G). Expression of this nuclease-dead construct did not rescue the lethality when expressed with the *69B-GAL4* driver (supplementary material Fig. S1).

**Fig. 2.**
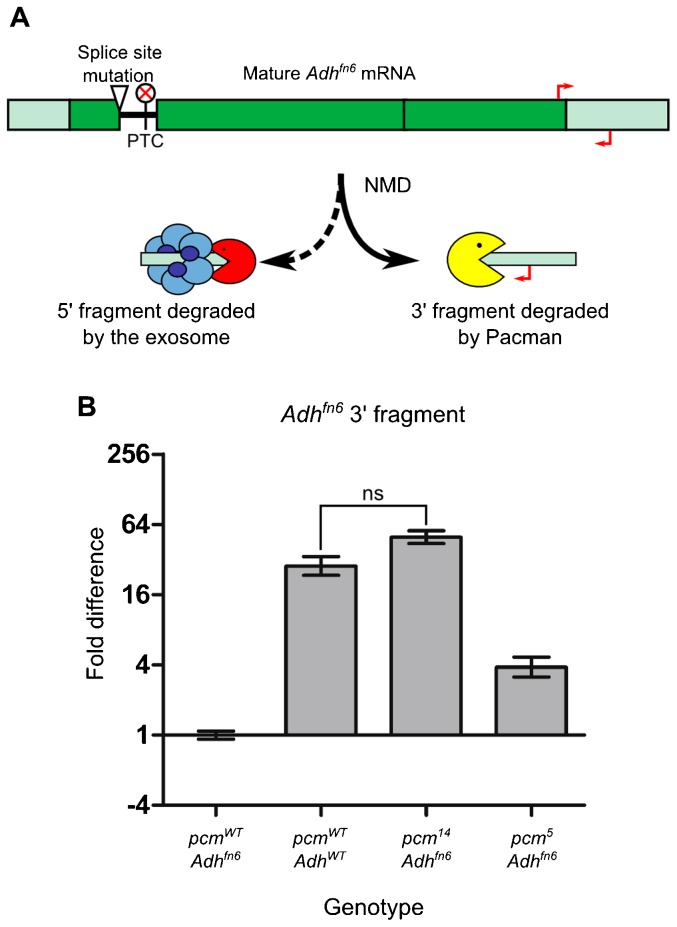
Estimation of the relative function of Pacman protein produced from the *pacman* alleles *in vivo*. (A) The *Adh^fn6^* allele of *Alcohol dehydrogenase* contains a splice site mutation which retains an in-frame stop codon in the intron. This causes the *Adh^fn6^* mRNA to undergo NMD, during which the 5′ fragment is degraded by the exosome and the 3′ fragment is degraded by Pacman. (B) To estimate the level of Pacman function in the *pcm^5^* and *pcm^14^* alleles, the level of the fragment degraded by Pacman was compared between lines containing the *Adh^fn6^* allele and either *pcm^5^* or *pcm^14^* (n≥11. p<0.001 for all comparisons unless indicated, ns = not significant. Error bars represent standard error).

To confirm that the *pcm^14^* allele had no enzymatic activity *in vivo*, we carried out a novel assay in whole larvae utilising the process of nonsense-mediated decay (NMD). NMD in *Drosophila* begins by endonucleolytic cleavage, creating two RNA fragments. It has been shown in cell culture that Pacman is required for degradation of the 3′ fragment (Gatfield and Izaurralde, 2004). Using an allele of *Alcohol dehydrogenase*, *Adh^fn6^*, which contains a premature termination codon (PTC) and is known to undergo NMD (Fig. 2A) (Benyajati et al., 1982; Brogna, 1999), we showed that the level of the 3′ fragment in *pcm^14^*; *Adh^fn6^* double mutants is not significantly different from the level of the undegraded transcript in wild-type larvae (Fig. 2B). This is congruent with the genetic data demonstrating that *pcm^14^* is a null allele (Fig. 1C,D). This also fits with previous findings that large C terminal deletions reduce the exoribonuclease activity to less than 10%, despite not affecting the catalytic domain (Page et al., 1998). The relative function of the *pcm^5^* allele was calculated (using ΔΔCT values) as 66.6% [(6−2)/(6−0)*100 = 66.6%;], which demonstrates that the catalytic activity of the Pacman protein translated from the *pcm^5^* allele is 66.6% functional. This fits in with previous findings that deletions removing the extreme C terminal of *pacman*, but not the SH3 domain, reduce the catalytic function to 65% that of wild type in *S. cerevisiae* (Page et al., 1998).

### *pcm^14^* mutant larvae have small L3 wing imaginal discs

**Fig. 3.**
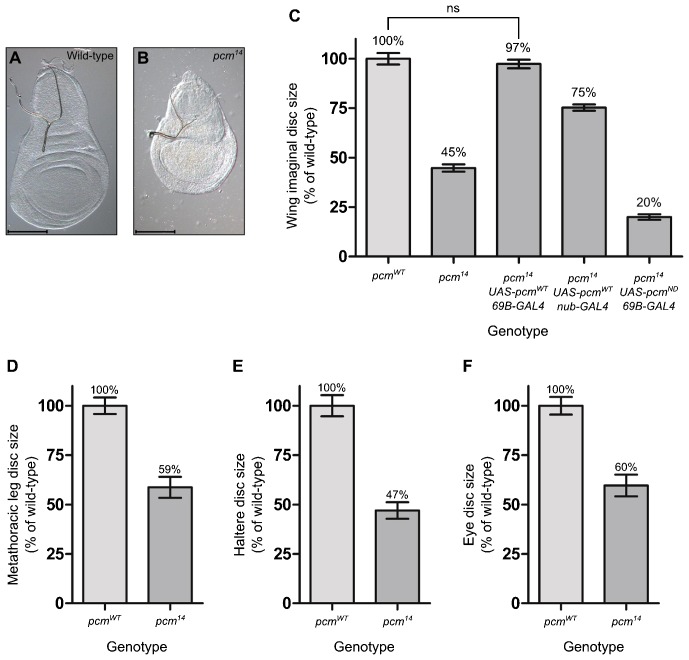
*pcm^14^* larvae have significantly smaller imaginal discs than wild-type larvae. (A,B) Representative wild-type and *pcm^14^* wing imaginal discs. Scale bar represents 100 µm. (C) The mean size of *pcm^14^* wing imaginal discs is 45% the size of wild type. This phenotype can be rescued by expressing a *UAS-pcm^WT^* construct throughout the wing imaginal disc cells using the *69B-GAL4* driver. Driving *UAS-pcm^WT^* expression with *nub-GAL4* partially rescues this phenotype to 75% the size of wild type. Expressing a *UAS-pcm^ND^* construct throughout the disc reduces the mean wing disc size to 20% of wild type (n≥31). (D) The mean size of *pcm^14^* metathoracic leg discs is 59% the size of wild type (n≥21), *pcm^14^* haltere discs (E) are 47% the size of wild-type (n≥21) and *pcm^14^* eye discs (F) are 60% the size of wild type (n≥16). p<0.001 for all comparisons unless indicated, ns = not significant, error bars represent 95% confidence limits.

**Fig. 4.**
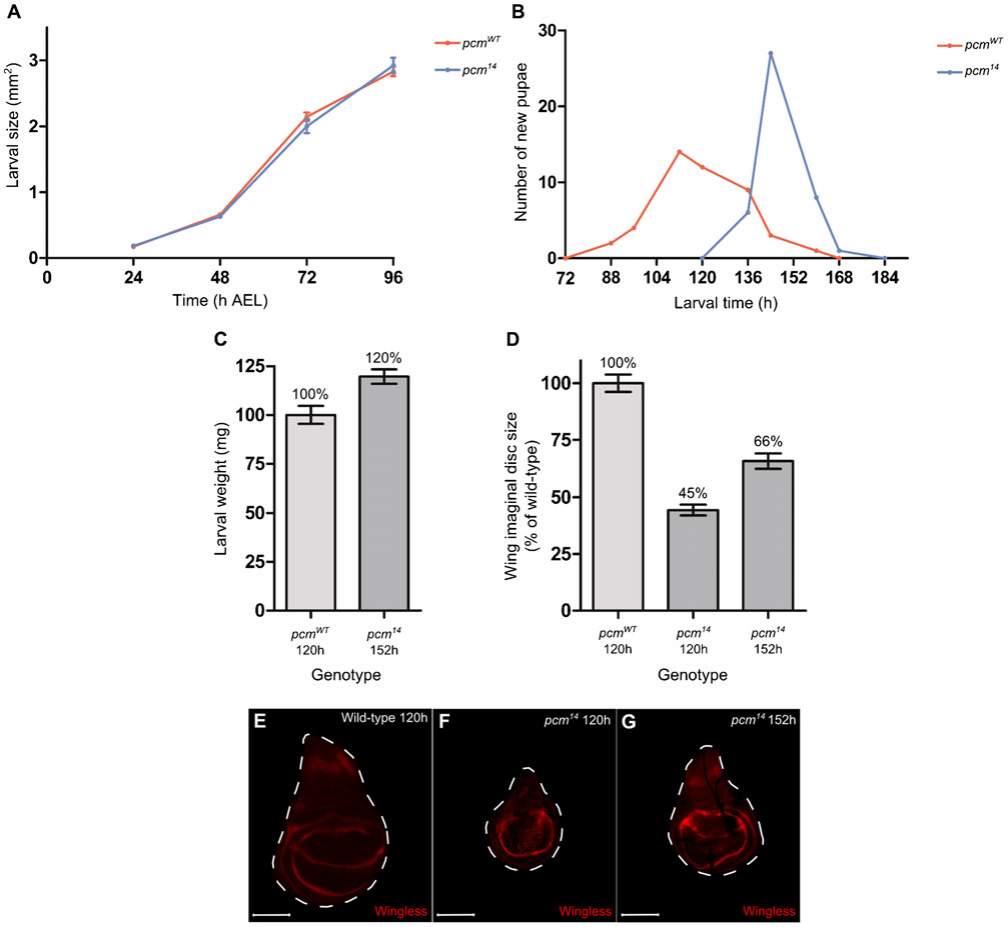
*pcm^14^* larvae are delayed in development. (A) The growth rate of *pcm^14^* larvae is not significantly different to wild type (n≥16). (B) Onset of pupariation of *pcm^14^* larvae is delayed by around 32 hours compared to wild type (n≥42). (C) The mean weight of *pcm^14^* larvae just prior to pupariation (152 hours) is 120% compared to the mean size of wild-type larvae just prior to pupariation (120 hours). (n≥35, p<0.0001). (D) During the extra 32 hours of development that *pcm^14^* larvae undergo, the size of the wing imaginal discs increases from 45% to 66% the size of wild type. (n≥30, p<0.001 for all comparisons). Error bars represent 95% confidence limits. (E–G) Wing imaginal disc development in *pcm^14^* larvae is morphologically delayed by 32 hours as determined by Wingless staining. (E) Wild-type wing imaginal disc at 120 hours displaying the correct pattern of Wingless. (F) *pcm^14^* wing imaginal disc at 120 hours does not display the correct pattern of expression for this time point. The expression is more diffuse throughout the wing pouch and does not contain the two rings of expression surrounding the wing pouch. (G) *pcm^14^* wing imaginal disc at 152 hours displaying the pattern of Wingless expression seen in wild type at 120 hours. Scale bars represent 100 µm.

The *pcm^5^* hypomorphic allele results in imaginal discs that are 18% smaller than those of wild type and also wings that are 16% smaller in viable adults (Jones et al., 2013). As expected, the *pcm^14^* wing imaginal discs are substantially smaller than *pcm^5^* discs, at 45% the size of wild type (Fig. 3A,B). We showed that the small wing disc size of the *pcm^14^* mutants is a consequence of lack of expression of wild-type *pacman* in the wing discs by rescuing the phenotype using the *69B-GAL4* driver to drive expression of *UAS-pcm^WT^* over the entire wing disc (Fig. 3C). The *nub-GAL4* driver, which drives expression only in the wing pouch, in combination with *UAS-pcm^WT^* rescues the wing imaginal disc size to 75% the size of wild type, demonstrating that Pacman is required throughout the wing disc (Fig. 3C). This rescue is dependent on the exoribonuclease activity of Pacman as no rescue was observed when the nuclease-dead *pacman* (*UAS-pcm^ND^*) construct was expressed with the *69B-GAL4* driver and actually reduced the overall size significantly to 25% the size of wild type, suggesting a dominant negative effect (Fig. 3C). In order to determine whether this growth phenotype was specific to the wing imaginal discs or whether all imaginal discs were affected, we measured the size of the metathoracic leg, haltere and eye imaginal discs in wild-type and *pcm^14^* L3 larvae (Fig. 3D–F). These other imaginal discs were smaller than those of wild type demonstrating that this phenotype is not specific to the wing imaginal discs. We chose to concentrate our investigations on the role of Pacman in the wing imaginal disc, as the development of this disc is better characterised than that of other discs and is also where the phenotypes are most apparent. The small size of the wing imaginal discs in *pcm^14^* mutants is not due to a decrease in the overall size of the larvae as the growth rates of wild-type and mutant larvae are identical, suggesting that Pacman specifically affects the growth of the imaginal discs (Fig. 4A). We also observed that *pcm^14^* larval development is significantly delayed, in that the majority of pupariation occurred at 136 hours after egg lay (AEL) in wild type compared to 168 hours AEL in *pcm^14^* mutants (Fig. 4B). During this extra 32 hours of development the *pcm^14^* larvae continue to feed and grow as *pcm^14^* larvae weighed significantly more than wild-type larvae immediately prior to pupariation (Fig. 4C). In addition, the size of the wing imaginal discs also increased from 45% to 66% the size of wild-type during the extended 32 hours of *pcm^14^* development (Fig. 4D). By staining the *pcm^14^* discs for the Wingless protein, which is expressed in a distinct pattern in the mature wild-type L3 wing imaginal disc at 120 hours (Fig. 4E) (Couso et al., 1994) we showed that the wing discs at 120 hours had an incomplete pattern of Wingless expression (Fig. 4F) and were therefore immature, whereas at 152 hours, when the majority of the mutant larvae are about to pupate, the pattern of Wingless expression was similar to wild type (Fig. 4G). Therefore *pcm^14^* wing discs are delayed in both growth and differentiation.

**Fig. 5.**
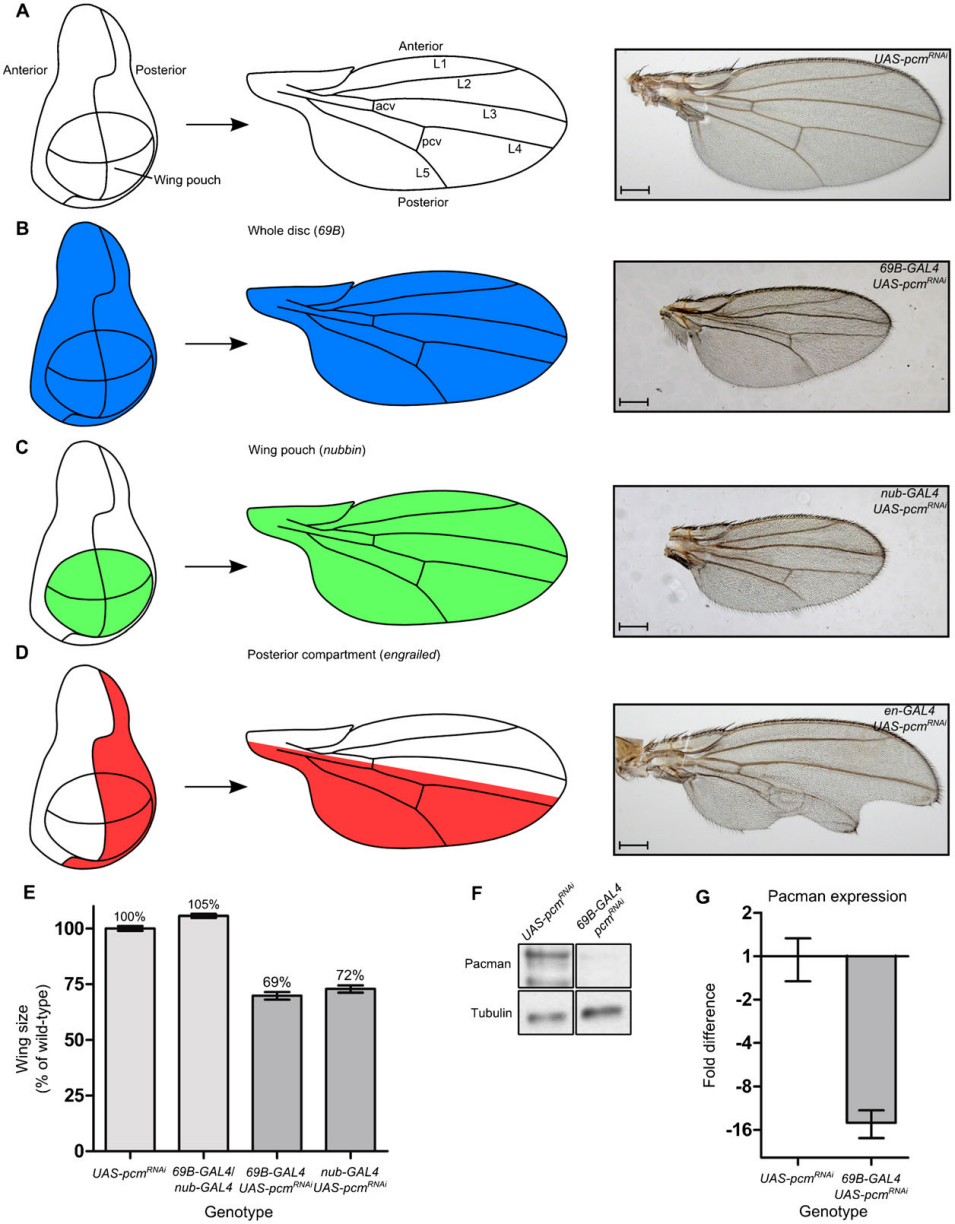
Knockdown of *pacman* using RNAi specifically within the wing imaginal discs results in smaller wings and wing vein defects. (A) Diagrammatic representation of a wing imaginal disc fate map forming the adult wing and a typical wild-type wing. (B) Knockdown of *pacman* throughout the wing imaginal discs using *69B-GAL4* leads to smaller wings and wing vein defects including loss of the anterior cross vein (94%), shortened L5 (8%) and shortened posterior cross vein (6%); (n = 49). (C) Knockdown of *pacman* specifically in the wing pouch using *nub-GAL4* also leads to smaller wings and also wing vein abnormalities such as loss of the anterior cross vein (100%), and a shortened L5 vein (40%); (n = 33). (D) Knockdown of *pacman* specifically in the posterior compartment of the wing imaginal discs using *en-GAL4* leads to defects in the posterior of the adult wing, The most common phenotypes recorded were blisters (66%), notches/loss of tissue (62%), and wing veins abnormalities, such as shortened L5 (19%) or branching of the posterior cross vein (13%) (n = 818). Scale bar represents 200 µm. (E) Wing sizes in *69B*-*GAL4*/*UAS-pcm^RNAi^* and *nub-GAL4*/*UAS-pcm^RNAi^* wings compared with their parental controls. (n≥33, error bars represent 95% confidence limits. p<0.001 for all comparisons except between *69B-GAL4/UAS-pcm^RNAi^* and *nub-GAL4/UAS-pcm^RNAi^* where p<0.05.). (F,G) Western blotting experiments to quantitate Pacman expression in *69B-GAL4*/*UAS-pcm^RNAi^* wing imaginal discs, show that it is knocked down almost 16 fold when compared to the *UAS-pcm^RNAi^* parental control. (n = 3, error bars represent standard error.)

We also tested whether the reduced growth of the wing imaginal discs in *pcm^14^* mutants is due to a lack of functional Pacman protein in the wing imaginal disc cells themselves rather than a consequence of the whole larva failing to develop correctly and signalling to the wing imaginal discs to delay their development. Knockdown of *pacman* expression in specific domains of the wing disc using various GAL4 drivers resulted in loss of tissue in the corresponding domains of the adult wing (Fig. 5). Therefore Pacman would appear to be required cell autonomously.

### Populations of *pcm^14^* wing imaginal disc cells are smaller than populations of wild-type wing imaginal disc cells

**Fig. 6.**
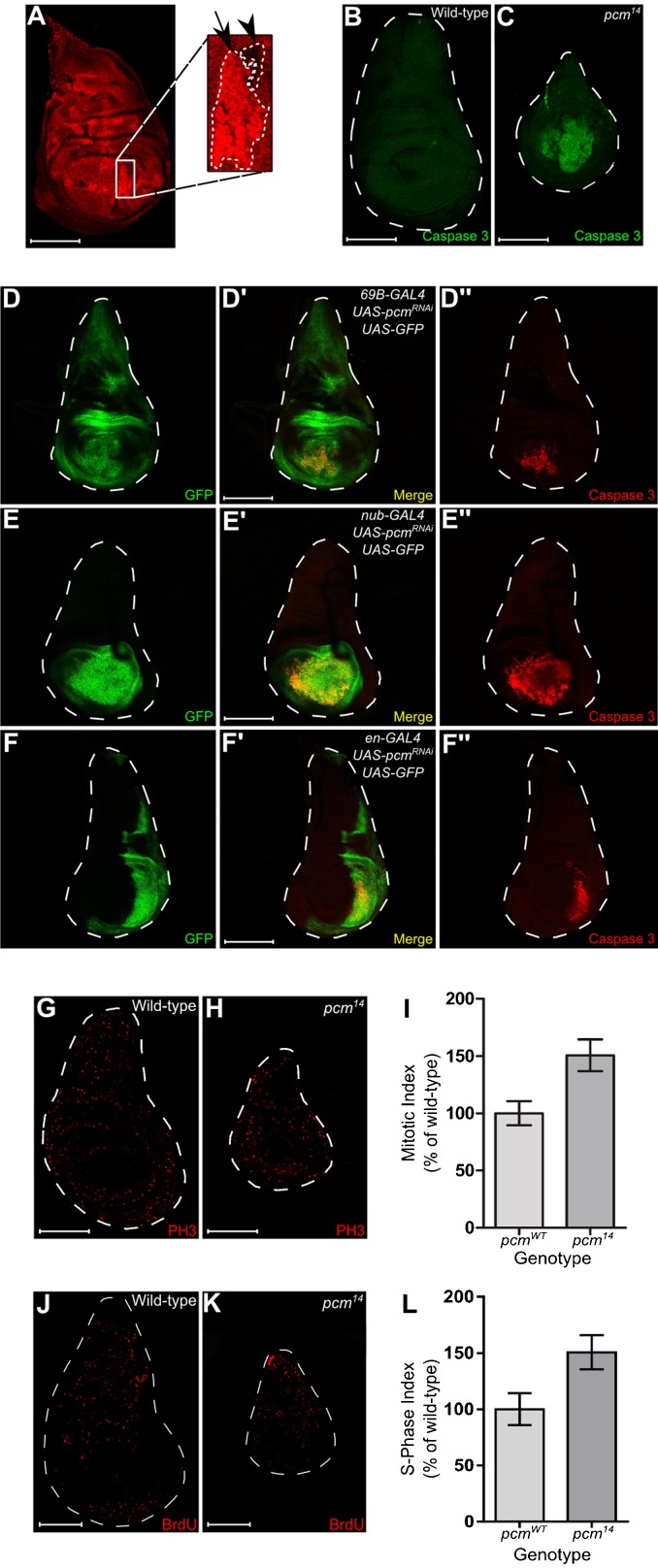
Populations of *pcm^14^* cells have reduced growth compared to populations of wild-type cells as a result of an increase in apoptosis. (A) Mosaic analysis was performed to directly compare growth rates between wild-type and *pcm^14^* mutant cells. Background cells are *pcm^WT^*/*pcm^14^*, cells increased in fluorescence are *pcm^WT^*/*pcm^WT^* and cells with no fluorescence are *pcm^14^*/*pcm^14^*. Mutant clones (arrowhead) were significantly smaller than their wild-type twin spots (arrow) (n = 25). The boundaries of wild type and mutant clones are marked by a white dashed line. Wild-type and *pcm^14^* wing imaginal discs were stained with an antibody for activated Caspase 3 which labels cells undergoing apoptosis. Cells throughout the wing pouch of the *pcm^14^* discs (C) were undergoing apoptosis which is not seen in wild type (B) (n≥39). Knockdown of *pacman* using RNAi driven by (D–D″) *69B-GAL4*, (E–E″) *nub-GAL4* or (F–F″) *en-GAL4* caused apoptosis to occur specifically within the wing pouch region of the disc. GFP fluorescence indicates regions of the disc expressing the *UAS-pcm^RNAi^* construct and Caspase 3 staining indicates regions of the disc where cells are undergoing apoptosis (n≥6). Note that these imaginal discs express *UAS-GFP* as well as *UAS-pcm^RNAi^* under the control of the relevant driver. (G,H) Wild-type and *pcm^14^* wing imaginal discs were stained with an antibody for phosphohistone H3 which labels cells in M phase. (I) A mitotic index was calculated by dividing the number of cells in M phase by the area of the disc. The mitotic index was increased in *pcm^14^* discs by 150% compared with wild type (n≥14, p<0.001. Error bars represent 95% confidence limits). BrdU incorporation of cells at S-phase within wild-type (J) and *pcm^14^* wing imaginal discs (K) was visualised using an antibody for BrdU. (L) A S-phase index was calculated by dividing the number of cells in S-phase by the area of the disc. The S-phase index was increased in *pcm^14^* discs by 154% compared with wild type (n≥8 p<0.01. Error bars represent 95% confidence limits). Scale bars represent 100 µm.

To confirm the cell autonomous requirement for Pacman in wing disc cells we used mosaic analysis where clones of cells with a mutant or wild-type homozygous genotype are induced in a heterozygous background by mitotic recombination. Populations of *pcm^14^*/*pcm^14^* cells and *pcm^+^/pcm^+^* cells were induced in a background of *pcm^14^*/*pcm^+^* cells, using the FLP/FRT system. If the *pcm^14^* mutation does result in a reduced growth rate, then a reduction in size of the mutant clones compared to their wild-type twin spots would be expected (Neto-Silva et al., 2009). When mitotic recombination was induced 48 hours AEL, mutant clones are clearly visible throughout the disc, alongside the wild-type twin spots, which were larger in size, showing that wild-type cells do indeed have a growth advantage over *pcm^14^* cells (Fig. 6A). This result also demonstrates a cell autonomous requirement for Pacman for correct growth.

### Loss of Pacman induces ectopic apoptosis in the wing pouch region of wing imaginal discs

The smaller size of the *pcm^14^* wing imaginal discs could be due to an increase in apoptosis, a decrease in cell division, or a combination of both. To determine whether there was an increase in apoptosis in *pcm^14^* wing imaginal discs compared to wild type, discs were stained with an anti-activated Caspase 3 antibody, which stains cells undergoing apoptosis. In the *pcm^14^* wing imaginal discs, large groups of cells in the wing pouch undergo apoptosis (Fig. 6C). This does not occur in the wild-type discs (Fig. 6B). Therefore loss of *pacman* appears to induce apoptosis in the wing pouch which could account for the small size of the wing discs.

To determine whether loss of *pacman* induces apoptosis only in the wing pouch area of the disc we knocked down *pacman* in different regions of the disc using the GAL4-UAS system and monitored apoptosis activity by anti-activated Caspase 3 staining. As can be seen from Fig. 6D, knockdown of *pacman* over the entire disc using the *69B-GAL4* driver results in apoptosis only in the wing pouch (Fig. 6D″). In the case of the *engrailed-GAL4* driver, which drives expression in the posterior half of the disc, apoptosis also occurs only in the posterior part of the wing pouch (Fig. 6F″) These results demonstrate that the ectopic apoptosis is specific to the wing pouch region of the disc during the L3 stage of development.

### The *pcm^14^* mutation results in compensatory proliferation of wing imaginal disc cells

The results of the mosaic analysis experiment above, where *pcm^14^*/*pcm^14^* mutant clones were smaller than *pcm^+^*/*pcm^+^* clones could also be explained by a decrease in cell division. To test this, the rate of cell division in the *pcm^14^* imaginal discs, compared to wild type, was monitored by staining the discs with an anti-phosphohistone H3 antibody, which detects cells undergoing mitosis. The nuclei undergoing division were counted and the mitotic index (the number of cells in M phase/area of disc) was calculated. Surprisingly, these results suggest that the rate of cell division in mutant discs is 51% higher than in wild-type discs (Fig. 6G–I). To assess whether the cells stained using phosphohistone H3 are blocked in M phase, rather than undergoing proliferation, we also used bromodeoxyuridine (BrdU) incorporation to identify cells undergoing DNA synthesis (i.e. S-phase) (Schubiger and Palka, 1987). This data shows that cell division is 54% higher in mutant discs than the control discs (Fig. 6J–L), confirming the phosphohistone H3 staining results. Therefore, these results strongly suggest that cells within mutant discs are undergoing compensatory proliferation (Fan and Bergmann, 2008a; Martín et al., 2009) in an attempt to overcome the increased rate of apoptosis. Nevertheless, the smaller size of the mutant discs, even after the extended period of larval development, means that this compensatory proliferation is unable to counteract the increased levels of apoptosis.

### Inhibition of apoptosis rescues the *pcm^14^* wing imaginal disc phenotypes

**Fig. 7.**
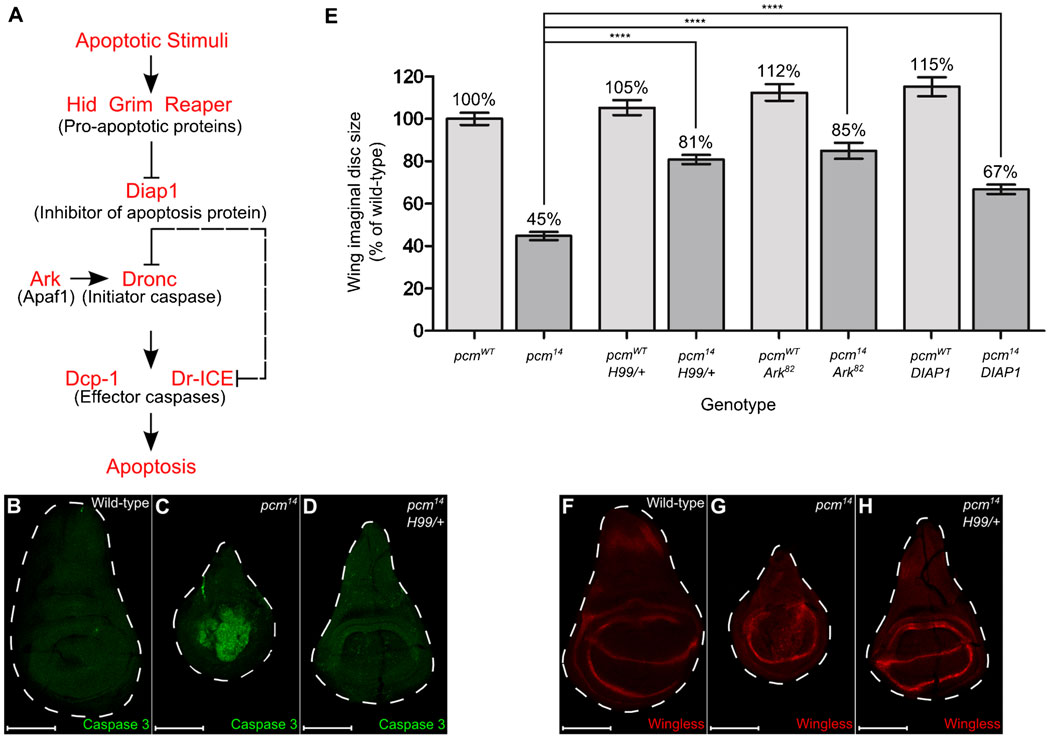
Inhibiting apoptosis partially rescues *pcm^14^* phenotypes. (A) A diagrammatic representation of the apoptosis pathway in *Drosophila*, indicating the main proteins involved in triggering apoptosis in response to an apoptotic stimuli. (B–D) Inhibiting apoptosis in *pcm^14^* wing imaginal discs was achieved by crossing in the *Df(3L)H99* deletion (which deletes pro-apoptotic genes *hid*, *grim* and *reaper*) as a heterozygote. The presence of *Df(3L)H99* reduced the amount of Caspase 3 staining and increased the size of the *pcm^14^* wing discs (compare C with D) (n = 13). (E) Inhibiting apoptosis partially rescues the size of *pcm^14^* wing imaginal discs. Reducing the copy number of *reaper*, *hid* and *grim* from 2 to 1 using the *Df(3L)H99* deletion as a heterozygote (*pcm^14^*;;*Df(3L)H99*/+) rescues the wing disc size from 45% to 81% (n≥31). Use of the *Ark^82^* allele as a homozygote rescues wing disc size from 45% to 85% that of wild type, showing that the adaptor protein Ark is required for much of the *pcm^14^* induced apoptosis (n≥19). Overexpression of the Inhibitor of apoptosis protein DIAP1 using the *69B-GAL4* driver (*pcm^14^*;*GAL80_ts_*/+;*69B-GAL4*/*UAS-DIAP1)* rescues the size of *pcm^14^* discs from 45% to 67% that of wild type, confirming that Pacman is acting through the pathway in A (n≥19) ****p<0.0001. Inhibiting apoptosis in the *pcm^WT^* background increases wing disc size to 105% using the *Df(3L)H99* deletion (p<0.05), 112% using the *Ark^82^* homozygous mutation (p<0.0001) and 115% using the *UAS-DIAP1* (p<0.0001). Error bars represent 95% confidence limits. (F–H) Inhibiting apoptosis rescues the delay in wing imaginal disc development as determined by Wingless staining at 120 hours (compare G with H) (n = 13). Scale bars represent 100 µm.

In *Drosophila*, the key activators of the caspase-induced apoptosis pathway are the Reaper, Hid and Grim proteins (Steller, 2008; Xu et al., 2009) (Fig. 7A). These are located adjacent to each other on the chromosome arm 3L and are removed by the deficiency *Df(3L)H99* (Chen et al., 1996; Grether et al., 1995; White et al., 1994). To determine whether Pacman acts through this pathway, we crossed *Df(3L)H99* into the *pcm^14^* mutant as a heterozygote, and analysed the effects on wing imaginal discs. Fig. 7D shows that apoptosis in *pcm^14^;;Df(3L)H99/+* imaginal discs is significantly reduced compared to *pcm^14^* discs (Fig. 7C and Fig. 6C) and is similar to that of wild type (Fig. 7B and Fig. 6B). Furthermore, the size of the imaginal discs is partially rescued to 81% the size of wild-type discs compared to 45% the size for *pcm^14^* discs (Fig. 7E). Staining *pcm^14^;;Df(3L)H99/+* wing discs at 120 hours AEL shows the characteristic Wingless expression pattern (Fig. 7H) as seen in wild-type discs (Fig. 7F and Fig. 4E). Therefore the delay in development of *pcm^14^* discs is also rescued by reduced expression of the caspase pathway genes.

In order to determine whether the adapter protein Ark (Apaf-1) (Fig. 7A) is required for the ectopic apoptosis in *pcm^14^* wing imaginal discs, Ark activity was completely removed using the null *Ark^82^* mutation as a homozygote (Akdemir et al., 2006). This significantly rescued the size of *pcm^14^* wing imaginal discs from 45% to 85% the size of wild type, suggesting that Ark is required for most but not all of the apoptosis occurring in these discs. This supports recent findings (Kang and Bashirullah, 2014) showing that apoptosis induced prior to late L3 is independent of apoptosome formation. In addition, overexpressing the inhibitor of apoptosis protein DIAP1 (Fig. 7A) in wing imaginal disc cells, rescued the size of *pcm^14^* wing imaginal discs to 67% the size of wild type. This was achieved using the *UAS-DIAP1* construct under *69B-GAL4* control. These results demonstrate that Pacman is indeed acting through the pathway depicted in Fig. 7A and that it is acting upstream of the Hid, Reaper and Grim proteins. Inhibiting apoptosis in a wild-type background increases wing imaginal disc size by 5% (*Df(3L)H99*/*+*), 12% (*Ark^82^*) or 15% (*UAS-DIAP1*), which is to be expected as it has been shown that there are low levels of apoptosis during normal wing imaginal disc development (Milán et al., 1997).

### *hid* and *reaper* are post-transcriptionally upregulated in *pcm^14^* wing imaginal discs

**Fig. 8.**
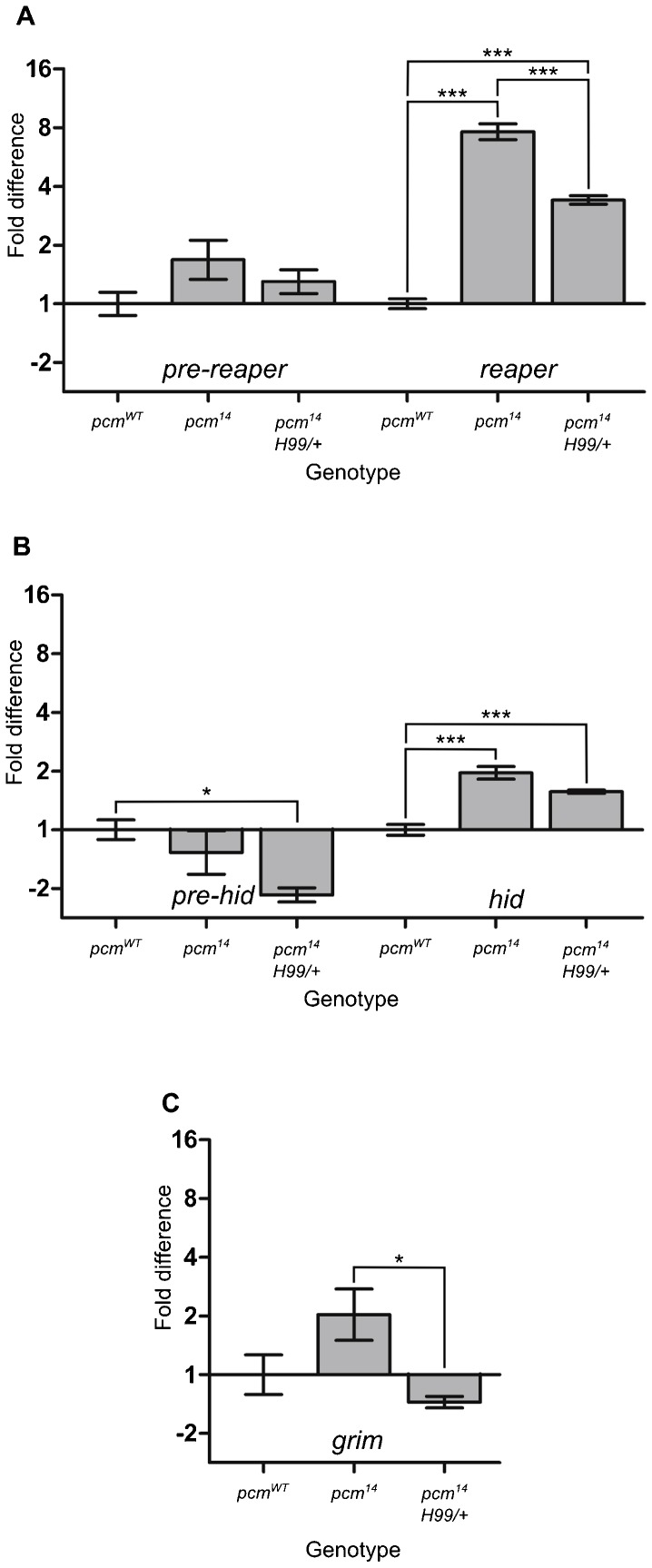
*reaper* and *hid* are post-transcriptionally upregulated in *pcm^14^* wing imaginal discs as determined by qRT-PCR. (A) Levels of *reaper* mRNA increase 7.8-fold in *pcm^14^* mutant wing imaginal discs compared with wild type whereas levels of *pre-reaper* RNA are not significantly different. The increase in mature *reaper* RNA is halved to 3.4-fold in *pcm^14^*;;*Df(3L)H99/+* mutant wing imaginal discs, where there is only one copy of the *reaper*, *hid* and *grim* genes. (n = 6 for wild type and *pcm^14^* and n = 5 for *pcm^14^*;;*Df(3L)H99*. ***p<0.001. Error bars represent standard error). (B) Levels of *hid* mRNA increase 2-fold in *pcm^14^* mutant wing imaginal discs compared with wild type, whereas levels of *pre-hid* RNA do not differ significantly. The levels of mature *hid* are reduced in *pcm^14^*;;*Df(3L)H99*/*+* wing imaginal discs, although this difference only showed statistical significance if no correction for multiple comparisons was performed (i.e. if a *t*-test was used to compare *hid* levels in *pcm^14^* and *pcm^14^*;;*Df(3L)H99*, rather than an ANOVA). (n = 6 for wild type and *pcm^14^* and n = 5 for *pcm^14^*;;*Df(3L)H99*. ***p<0.001 and *p<0.05. Error bars represent standard error). (C) Levels of *grim* mRNA are not significantly different between wild-type and *pcm^14^* wing imaginal discs. *grim pre-mRNA* could not be reliably detected. (n = 6 for wild type and *pcm^14^* and n = 5 for *pcm^14^*;;*Df(3L)H99*. *p<0.05. Error bars represent standard error). *RpL32* (*Rp49*) was used for normalisation.

Pacman is an exoribonuclease that degrades mRNAs. We therefore hypothesised that Pacman could specifically target *hid*, *grim* and *reaper* mRNA with the consequence that the loss of Pacman would result in increased levels of these mRNAs. To test this, we used quantitative TaqMan qRT-PCR to measure the levels of *hid*, *grim* and *reaper* mRNAs in *pcm^14^* mutant wing imaginal discs. As can be seen from Fig. 8, *reaper* mRNA is increased by 7.8-fold in *pcm^14^* wing imaginal discs compared to wild type whereas *hid* mRNA is increased by 2-fold. The levels of *grim* mRNA were very low and variable and showed no significant difference to those of wild type. In the rescued wing imaginal discs (*pcm^14^;;Df(3L)H99/+*) *reaper* and *hid* mRNA levels were intermediate between *pcm^14^* discs and wild-type discs, as expected.

If Pacman is involved in the degradation of *reaper* and *hid* mRNAs, then we would expect these mRNAs to increase at the post-transcriptional level but not at the transcriptional level in the *pcm^14^* mutants. To test this, we used primer-probe assays designed to detect *reaper* and *hid* pre-mRNAs, but not their mRNAs (supplementary material Fig. S3). These experiments show that *reaper* and *hid* pre-mRNA levels in the *pcm^14^* mutant are not significantly different from that of the wild-type control (p = 0.0808 and 0.3634 respectively; Fig. 8A,B). Therefore *reaper* and *hid* mRNAs show a significant increase at the post-transcriptional level but not the transcriptional level suggesting that Pacman normally targets these mRNAs for degradation.

To determine whether the upregulation of *reaper* and *hid* mRNA in *pacman* mutants is a specific effect, rather than a general effect on many RNAs, we also analysed the effect of the *pacman* null mutation on the levels of other RNAs. TaqMan qRT-PCR experiments using primer-probe assays for the cell cycle mRNAs *string* (*cdc25*), *CyclinD* and *CyclinE* mature mRNA showed no differences in expression levels between *pcm^14^* and wild-type wing imaginal discs (supplementary material Fig. S4). This is consistent with a specific effect of Pacman on *reaper* and *hid* mRNAs *in vivo*.

An increase of *reaper* and *hid* mRNA in *pcm^14^* mutants would normally mean that Reaper and Hid proteins are also increased. We were, however, unable to test this directly using western blotting as the antibodies available to us gave non-specific bands or no bands at all. Nevertheless, the phenotypic effects we see in *pcm^14^* mutants, or as a result of *pacman* knockdown, and the genetic interactions of these mutants with other genes in the caspase pathway, are entirely consistent with protein being expressed from the increased *reaper* and *hid* mRNAs. Taken together, our results therefore show that Pacman can regulate apoptosis in *Drosophila* wing imaginal discs and that this regulation mainly takes place at the post-transcriptional level.

## DISCUSSION

Apoptosis is a key process in developmental pathways and also in cancer. In the present study, we have generated a null mutation in *pacman* (*pcm^14^*) and used this to show that Pacman can control apoptosis in wing imaginal discs by regulating levels of *hid* and *reaper* mRNAs. Use of the *Df(3L)H99* deletion, which removes one copy of the *hid*, *grim* and *reaper* genes, largely rescues the effect of the *pcm^14^* mutation on growth of the wing imaginal discs and on developmental timing. However, the *Df(3L)H99* deletion (*Df(3L)H99/+*) does not rescue the lethality of the *pcm^14^* mutation. This suggests that there may be other targets of Pacman that are misregulated in *pcm^14^* larvae or pupae.

According to the data presented in Fig. 6C, the mutant wing discs are proportionately reduced in size, even though the majority of apoptosis occurs in the wing pouch. Our data also show that Pacman is expressed over the entire disc (supplementary material Fig. S2) and *pcm^14^*/*pcm^14^* mutant clones are smaller than their wild-type twin spots throughout the disc (Fig. 6A). It is possible that apoptosis is occurring throughout the disc in earlier stages of development but is restricted to the wing pouch during L3. Our data is consistent with other studies reporting that cells within the wing pouch are particularly sensitive to apoptosis (Kang and Bashirullah, 2014), perhaps due to expression of particular apoptotic regulators in that region (Bejarano et al., 2010). The co-ordinate growth of the wing disc, even though apoptosis is occurring in a particular region of the disc, is likely to be due to long range signalling via morphogens which control overall patterning and growth of the wing disc. For example, Decapentaplegic (Dpp), a bone morphogenetic protein (BMP) functions as a long range morphogen to control patterning and growth (Vuilleumier et al., 2010). Furthermore, the Aegerter-Wilmsen model which explains how growth is constant throughout the disc suggests that growth of the peripheral cells within the disc is caused by stretching of the cells as a result of growth at the centre of the disc (Aegerter-Wilmsen et al., 2007; Aegerter-Wilmsen et al., 2012). Therefore, reduced growth at the centre of the disc, caused by apoptosis specifically in the pouch, is likely to cause reduced growth of the whole disc.

Our results also show that the *pcm^14^* mutation induces cell proliferation as well as apoptosis. Apoptosis-induced compensatory proliferation is known to occur to maintain tissue homeostasis so that damaged tissues can be replaced allowing the organ to maintain its normal size (Fan and Bergmann, 2008a; Martín et al., 2009). In *Drosophila*, this occurs via the initiator caspase Dronc which induces compensatory proliferation as well as apoptosis (Fan and Bergmann, 2008b; Huh et al., 2004; Kondo et al., 2006; Wells et al., 2006). Since Dronc is activated by Hid and Reaper, the increase in *hid* and *reaper* mRNA in *pcm^14^* cells is consistent with increased activity of Dronc. Nevertheless, the 51%–54% increase in cell division in the *pcm^14^* wing imaginal discs is insufficient to compensate for the concurrent increase in apoptosis because the wing discs fail to develop and differentiate, leading to death of the pupa. This failure of the wing discs to regenerate could be explained by there being prolonged apoptosis in the *pcm^14^* wing imaginal discs, whereas other experiments have induced a pulse of apoptosis, allowing time for the wing disc to recover (Milán et al., 1997; Pérez-Garijo et al., 2004).

The above results are consistent with *reaper* and *hid* being translated from the upregulated *reaper* and *hid* transcripts in *pcm^14^* mutants. This would imply that these transcripts are both capped and polyadenylated. Biochemical analyses have shown that the less structured C-terminal domain of Pacman/Xrn1 includes short sections of conserved amino acids which bind co-factors such as the decapping protein Dcp1. Dcp1 associates with the decapping enzyme Dcp2, therefore coupling decapping to 5′-3′ degradation (Braun et al., 2012). In *pcm^14^* cells where no Pacman is present, decapping would therefore be expected to be impaired, which is consistent with our data. The alternative and/or additional hypothesis is that *reaper* and *hid* are being translated in a cap independent manner. Indeed the 5′ UTRs of these genes have been shown to contain functional Internal Ribosome Entry Sites (IRES) and are still able to undergo translation in cells in which cap dependent translation is impeded (Hernández et al., 2004; Vazquez-Pianzola et al., 2007).

The above molecular mechanisms also are consistent with the “dominant negative” effect seen when we express the nuclease-dead version of Pacman in a *pcm^14^* mutant background. In *Drosophila* tissue culture cells, over-expression of catalytically inactive Pacman inhibited both decapping and degradation of a reporter RNA leading to an accumulation of capped fragments (Braun et al., 2012). Therefore the dominant negative effect could result from the sequestration of the Decapping protein Dcp1 together with lack of exonuclease activity. Expressing a “nuclease dead” Pacman in *pcm^14^* cells would not rescue any exoribonuclease activity but could impair decapping further. Our results therefore support the model (Jones et al., 2012) that Pacman/Xrn1 normally assembles a complex of 5′-3′ degradation factors including Dcp1 to provide a multicomponent complex which decaps and then degrades specific RNAs in a 5′-3′ direction.

Our data, using natural tissue rather than immortalised tissue culture cells, supports the idea that there is a network of RNA-protein interactions contributing to apoptosis and proliferation. This idea is supported by work on the deadenylases Ccr4a and Ccr4b which can affect cell survival in MCF7 human breast cancer cells (Mittal et al., 2011). Further, the RNA-binding protein HuR (homologue of Elav in *Drosophila*) has recently been shown to be cleaved in HeLa cells during caspase-mediated apoptosis with the two cleavage fragments binding to and stabilising *caspase 9* mRNA, thus promoting apoptosis (von Roretz et al., 2013). Our data showing that the exoribonuclease Pacman is also involved in the control of apoptosis suggests a key role for the 5′-3′ degradation pathway in the regulation of apoptosis.

What are the mechanisms by which Pacman might be affecting the levels of mature *hid* and *reaper* mRNA? The simplest hypothesis is that Pacman is normally targeted to *hid* and *reaper* mRNA, resulting in degradation of these mRNAs. This targeting could be accomplished by specific RNA binding proteins and/or miRNAs binding to the 3′ UTRs of *hid* and *reaper* mRNAs and directing them to the 5′-3′ degradation machinery (Eulalio et al., 2007; Jones et al., 2012; Nishihara et al., 2013). The 3′ untranslated regions of *hid* and *reaper* contain many predicted and validated miRNA binding sites for miRNAs (Brennecke et al., 2003; Brennecke et al., 2005; Ge et al., 2012; Hilgers et al., 2010; Ruby et al., 2007; Xu et al., 2004). For example, the miRNA *bantam* has been shown to bind to the 3′ UTR of *hid* mRNA, thus regulating its expression (Brennecke et al., 2003). In addition, *miR-2* is known to bind to the 3′ UTR of *reaper*, repressing its translation and directing it to P-body-like structures (Thermann and Hentze, 2007). A possible model to explain our results is that *reaper* and *hid* mRNAs are normally unstable because they are directed to the 5′-3′ degradation complex by miRNAs binding to their 3′ UTRs. In wild-type cells, these RNAs are rapidly decapped by decapping enzymes associated with Pacman and then degraded in a 5′-3′ direction. In the Pacman mutant, these mRNAs are not efficiently degraded because of the absence of Pacman. It is also possible that *reaper* and *hid* are particularly affected by loss of Pacman because the presence of IRES sequences within their 5′ UTRs (Hernández et al., 2004; Vazquez-Pianzola et al., 2007) means that these RNAs can be translated even if they are decapped. In a *pacman* mutant, these decapped RNAs may still be translated to produce Reaper and Hid protein. The exact mechanisms whereby Pacman regulates these mRNAs will require further research.

## Supplementary Material

Supplementary Material
