# Supplementary material for: Xrn1/Pacman affects apoptosis and regulates expression of hid and reaper
Source: Biol Open. 2015 Apr 2;4(5):649–60. doi: 10.1242/bio.201410199 (PMC4434816; doi:10.1242/bio.201410199)
Supplement: Supplementary Material [file supp_4_5_649__index.html]

Xrn1/Pacman affects apoptosis and regulates expression of hid and reaper — Xrn1/Pacman affects apoptosis and regulates expression of hid and reaper — Supplementary Material 

# Xrn1/Pacman affects apoptosis and regulates expression of *hid* and *reaper*

## bio.201410199 Supplementary Material

**Files in this Data Supplement:**

- Supplementary Material - Joseph A. Waldron et al. doi: 10.1242/bio.201410199
