# Supplementary material for: Xrn1/Pacman affects apoptosis and regulates expression of hid and reaper
Source: Biol Open. 2015 Apr 2;4(5):649–60. doi: 10.1242/bio.201410199 (PMC4434816; doi:10.1242/bio.201410199)
Supplement: Supplementary Material [file supp_bio.201410199_bio.201410199-s1.pdf]

Biology Open (2015) 000, 1–12 doi:10.1242/bio.201410199

Supplementary Material

Joseph A. Waldron et al. doi: 10.1242/bio.201410199

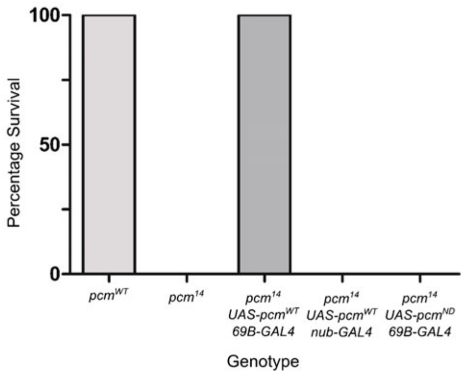

Fig. S1. The *pcm*[14] mutation causes lethality during the pupal stage of development. This lethality can be rescued by expressing a *UAS-pcm*[WT] construct with the *69B-GAL4* driver, but not with the *nub-GAL4* driver. Expression of *UAS-pcm*[ND] with *69B-GAL4* did not rescue lethality (n=60).

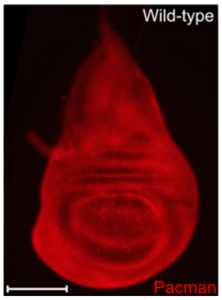

Fig. S2. Pacman is expressed throughout the wing imaginal disc in wild type. Scale bar represents 100 µm.

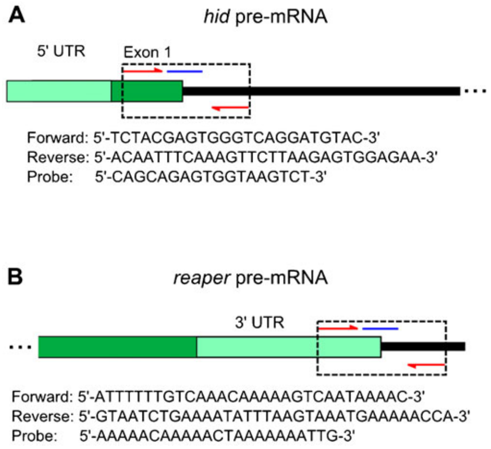

Fig. S3. Diagrammatic representation of the custom TaqMan primer/probe sets used to measure the pre-mRNAs of (A) *hid* and (B) *reaper*. Dashed box represents the 100 nt sequence submitted to Life Technologies' web-based Custom TaqMan Assay Design Tool. Actual sequences of primer/probes are listed.

Biology Open (2015) 000, 1–12 doi:10.1242/bio.201410199

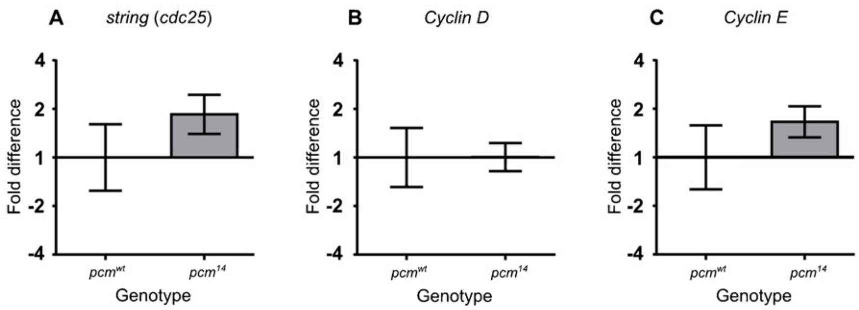

Fig. S4. The cell cycle genes *string* (*cdc25*), *Cyclin D* and *Cyclin E* are not significantly differentially regulated in *pcm*^{14} L3 wing imaginal discs compared to wild type. n=6 for *pcm*^{14} and n=7 for wild type. p=0.083 for *string,* p=0.984 for *Cyclin D* and p=0.094 for *Cyclin E*. Error bars represent standard error. *RpL32* (*Rp49*) was used for normalisation.
